# Facile Surface Treatment of 3D-Printed PLA Filter for Enhanced Graphene Oxide Doping and Effective Removal of Cationic Dyes

**DOI:** 10.3390/polym15020269

**Published:** 2023-01-04

**Authors:** Sung-Sil Park, Yun-Seok Lee, Seung-Woo Lee, Eveliina Repo, Tae-Hyun Kim, Yuri Park, Yuhoon Hwang

**Affiliations:** 1 Department of Environmental Engineering, Seoul National University of Science and Technology, Seoul 01811, Republic of Korea; 2 Department of Fine Chemistry, Seoul National University of Science and Technology, Seoul 01811, Republic of Korea; 3 Department of Separation Science, School of Engineering Science, LUT University, FI-53851 Lappeenranta, Finland; 4 Institute of Environmental Technology, Seoul National University of Science and Technology, Seoul 01811, Republic of Korea

**Keywords:** PLA scaffold, graphene oxide, acetone pre-treatment, 3D printing, adsorption

## Abstract

The structured adsorption filter material is one of the ways to enhance the practical applicability of powdered adsorbents, which have limitations in the real water treatment process due to difficulty in the separation process. In this study, three-dimensional (3D) printing technology was applied to prepare filter materials for water treatment processes. A 3D-printed graphene-oxide (GO)-based adsorbent is prepared on a polylactic acid (PLA) scaffold. The surface of the PLA scaffold was modified by subjecting it to strong alkaline or organic solvent treatment to enhance GO doping for realizing effective adsorption of cationic dye solutions. When subjected to 95% acetone treatment, the structural properties of PLA changed, and particularly, two main hydrophilic functional groups (carboxylic acids and hydroxyls) were newly formed on the PLA through cleavage of the ester bond of the aliphatic polyester. Owing to these changes, the roughness of the PLA surface increased, and its tensile strength decreased. Meanwhile, its surface was doped mainly with GO, resulting in approximately 75% methylene blue (MB) adsorption on the 3D-printed GO-based PLA filter. Based on the established optimal pretreatment conditions, a kinetic MB sorption study and an isotherm study were conducted to evaluate the 3D-printed GO-based PLA filter. The pseudo-second-order model yielded the best fit, and the MB adsorption was better fitted to the Langmuir isotherm. These results suggested that chemical adsorption was the main driver of the reaction, and monolayer sorption occurred on the adsorbent surface. The results of this study highlight the importance of PLA surface modification in enhancing GO doping and achieving effective MB adsorption in aqueous solutions. Ultimately, this study highlights the potential of using 3D printing technology to fabricate the components required for implementing water treatment processes.

## 1. Introduction

Since the industrial revolution, rapid population growth and urbanization have caused severe air, water, and soil pollution. Particularly, a wide range of hazardous pollutants, such as heavy metals, organics, and biotoxins, released from industrial processes and households have become major hazards for humans and other living organisms [1,2]. Thus, it is imperative to develop an effective separation technology for the removal of diverse pollutants from water.

One of the available technologies, the adsorption process, is considered a simple and easy process [3,4]. To enhance the adsorption process, an adsorbent with a large surface area, high pore volume, and appropriate functionalities is critical. In the past years, carbonaceous-based adsorbents, such as activated carbon, carbon nanotubes, and fullerene, that are characterized by high adsorption capacity and thermal stability, have been reported [5,6,7,8,9,10,11]. Recently, graphene oxide (GO), obtained through hydrophobic graphene functionalization via oxidation by using potassium permanganate (KMnO_4_), sulfuric acid (H_2_SO_4_), or perchloric acid (HClO_4_), has attracted tremendous attention from researchers because of its exceptional mechanical strength and unique thermal and electrical properties [12]. The presence of a substantial number of oxygen functional groups (e.g., carboxyl, carbonyl, epoxy, and hydroxyl groups) on the surface of carbon imparts a negative charge to GO and makes it hydrophilic [12]. Consequently, GO becomes a highly efficient adsorbent material for eradicating pollutants, such as heavy metals [13] and methylene blue (MB), a cationic dye, relative to activated carbon [12,14]. However, it is difficult to separate GO from the aquatic environment because of its small particle size and low specific weight. The need for an additional process, such as high-speed centrifugation or filtration, is one of the drawbacks of using the adsorption process as well as GO, despite the suitability of GO as an adsorbent for treating contaminated waters.

In this context, 3D printing, a novel technology, offers the possibility of fabricating objects with geometrically complex structures. Owing to its remarkable performance and time- and cost-effectiveness, 3D printing technology has been implemented using various printing materials to prepare 3D-printed materials in industries as diverse as the aerospace [15], biomedical [16], and food industries [17]. Particularly, polymer-based 3D printing is an efficient and single-step fabrication technique, and it allows for the design and fabrication of membrane modules, adsorbent monoliths, and photocatalytic composites intended for water treatment applications [18,19,20]. Fused deposition modeling (FDM) is a commonly used printing method in which thermoplastic materials are heated and injected. Polyamide (PA), polycarbonate (PC), polylactic acid (PLA), polyetheretherketone (PEEK), and polystyrene (PS) are among the thermoplastic materials that are commonly used in 3D printing [21,22,23]. However, the listed thermoplastic materials do not intrinsically possess the properties required to adsorb certain types of water pollutants. Therefore, further functionalization of 3D filters is necessary to ensure they can be used for water treatment. The fabrication of GO mixed polymer filament can also be prepared, but it has the limitation that the active sites of GO would be covered by the mixed polymer, which leads to lower adsorption efficiency. Moreover, the polymer rheology should be seriously considered when the mixed filament is used for 3D printing, which also requires additional time and effort. Therefore, in this study, it was aimed to dope the GO on the filter surface to maintain the intrinsic adsorption properties of GO while the 3D-structured filter is built.

Among the well-established surface-modification methods to control the properties of PLA, surface alkali hydrolysis treatment is a simple and convenient method for introducing two main reactive hydrophilic functional groups including carboxylic acids (-COOH) and hydroxyls (-OH), on the PLA surface through cleavage of the ester bond of the aliphatic polyester. For instance, sodium hydroxide (NaOH) is the most commonly used alkali for modifying the surface of PLA to improve its hydrophilicity [24]. Considering that a low-concentration aqueous NaOH solution is inadequate to cleave the ester bond on the PLA surface, a mixture of 0.25 M NaOH and ethanol was successfully used for this purpose [25], where ethanol served as a non-toxic and in-expensive co-treatment medium for assisting the hydroxide-driven nucleophilic attack on the ester bond, which is often facilitated by acetonitrile [25]. In addition, acetone treatment increased the hydrophilicity [26], although the NaOH treatment method was still proven to be a more effective approach. With respect to the PLA surface modification, there are still gaps in the knowledge of pre-treatment strategies affecting the structural and mechanical properties of PLA by the chemical etching process, which further enhances the uniform dispersion of GO on the surface of the polymer substrate. As such, this study investigates the surface modification strategies of PLA by applying two different types of pre-treatment chemicals (i.e., NaOH and acetone) to see the changes on the surface and mechanical properties of the PLA and to evaluate their effects on the GO dispersion for the fabrication of GO-based 3D-printed materials.

To this end, herein, this study describes the fabrication of a 3D-printed adsorbent on PLA, which is selected as the matrix phase because of its bio-based origin, bio-degradability, and well-established 3D-printability and flexibility in bio-composite fabrication processes. The pre-treatment conditions for PLA surface modification by using NaOH-based alkaline treatment or acetone-based organic solvent treatment are optimized. During the optimization process, acetone is chosen over other organic solvents because it is non-toxic and can be removed easily by rinsing. Moreover, it can be used to nucleophilically attack the ester bond for modifying the surface of PLA. The morphological and mechanical properties of PLA before and after surface modification are examined to determine the effect of surface modification on the adhesion between 3D-printed PLA and nano-scale GO. Finally, the adsorption capacity of the 3D-printed GO-based PLA filters for a cationic MB solution is tested. In sum, an effective pre-treatment approach for enhancing GO doping on PLA to design 3D-printed adsorbents is established. Moreover, this study demonstrates the feasibility of deploying the 3D-printed GO-based PLA filters in water treatment processes by using them to effectively remove cationic MB dye from aqueous solutions.

## 2. Materials and Methods

### 2.1. Materials

Commercial graphene oxide solution (FNG-A102, 10 wt.%) was obtained from Graphene-Tech Co. Ltd., Suzhou, China. MB was purchased from JUNSEI Co. Ltd. (Tokyo, Japan). Acetone (C_3_H_6_O, 99.8%) and sodium hydroxide standard solution (NaOH, 5N) were supplied by DAEJUNG Chemicals Co. (Siheung, Korea). PLA filaments (Snow White) with an average diameter of 1.75 mm were purchased from NatureWorks LLC, Fresno, CA, USA. All the chemicals were of analytical grade, and ultrapure water (deionized water; DI water) with a resistivity of 18.2 mΩ·cm (25 °C) was produced using a Milli-Q system (Synergy^®^, Merck Millipore, Burlington, MA, USA).

### 2.2. Fabrication of PLA Scaffold Using an FDM 3D Printer

A virtual model of the PLA scaffold was created in the NewCreatorK software environment (version 1.57.80; ROKIT Co., Seoul, Korea). The scaffold design was generated as image data, and the image data were directly converted into a surface representation in the Standard Triangulated Language (STL) format as input data for manufacturing. The scaffold was fabricated using an FDM 3D printer (AEP Ⅱ, ROKIT Co., Seoul, Korea).

**Figure 1:**
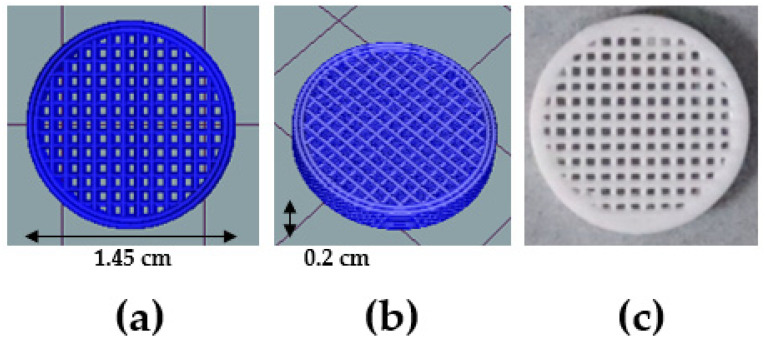
3D-printed PLA scaffold filter—(**a**,**b**) 3D design of the filter, (**c**) printed scaffold filter image.

When the nozzle and bed temperatures reached 200 °C and 50 °C, respectively, melted PLA filaments were extruded through a nozzle. Parallel PLA strands were deposited on the bed in a layer-by-layer fashion at a speed of 40 mm/min, and the infill was 40%. Square perforations were formed in the deposited structure through a 90° rotation of the meandering strand pattern. The scaffold, which was customized based on a quartz column (diameter 1.5 cm, Pyrex, Seoul, Korea) that is generally used in continuous water treatment experiments, was cylindrical [27]. The diameter and height of each printed scaffold filter were 1.45 cm and 0.2 cm, respectively, as shown in Figure 1. The Repetier Host software environment (ver.2.2.4; Hot-World GmbH & Co. KG, Willich, Germany) was used to check the images of the designed scaffold filter before production.

### 2.3. Production of GO/PLA Filter

The 3D-printed PLA scaffolds were chemically etched with NaOH (5N), as described by Schneider et al. [28]. Moreover, the printed PLA scaffolds were treated with different concentrations of acetone (acetone:DI water = 90–100:10–0, *v*/*v*) at ambient temperature to select the optimal pre-treatment condition compared to NaOH-based alkaline treatment. First, the PLA scaffolds were immersed in 10 mL of 5N NaOH for 4 h or in 10 mL of different concentrations of acetone for 24 h.

The PLA scaffolds chemically etched with NaOH or acetone (labeled as PLA-NaOH or PLA-acetone) were further doped with GO (10 wt.%) from a GO suspension in a sonicator (JP-080S, Skymen Cleaning Co., Shenzhen, China) for 1 min, and they were labeled as PLA-NaOH-GO and PLA-acetone-GO (GO/PLA), respectively. The GO-doped, pre-treated PLA filters were placed in a petri dish and dried in an oven at 60 °C for 60 min, where they were flipped over at intervals of 10 min. The dried filters were washed several times with DI water and sonicated (Power Sonic 410 Lv.3, Hwashin Tech. Co., Seoul, Korea) for 20 min to remove un-doped GO from the PLA scaffolds. After sonication, the GO/PLA filters were stored in DI water and used in the adsorption experiment. 

### 2.4. Characterization of GO/PLA Filter

The surface morphologies of the PLA, PLA-acetone, and GO/PLA filters were characterized using a high-resolution field emission scanning electron microscope (FE-SEM; SU8100, Hitachi High Technologies Co., Tokyo, Japan) operated at the accelerating voltage of 15 kV and emission current of 78 μA. Before SEM analysis, the samples were placed on the stub and coated with platinum (Pt) for 200 s. A Perkin Elmer Fourier transform infrared spectrometer (FT-IR; Spectra Two, Perkin Elmer Co., Madison, WI, USA) with attenuated total reflectance (ATR) was used to identify the functional groups of the PLA and GO/PLA in the range of 650–4000 cm^−1^ over a total of 8 scans at a resolution of 4 cm^−1^. A Thermo Scientific DX3i Raman spectroscope (a green laser with a wavelength of 532 nm; Thermo Scientific, Waltham, MA, USA) operated with the laser power of 5 mW and exposure time of 1 s was used to confirm the presence of GO on the surface of PLA. Thermogravimetric analysis (TGA) of the PLA and GO/PLA was performed using an SDT Q600 TGA (TA Instruments Inc., New Castle, DE, USA) operated at a ramp rate of 10 °C/min from room temperature to 800 °C in high-purity flowing nitrogen atmosphere. Approximately 12–15 mg of small slices of the PLA and GO/PLA samples were heated in an open platinum crucible.

Tensile tests were conducted by following the American Society for Testing and Materials (ASTM) D638 Standard Test Methods for Tensile Properties of Plastics [29] to investigate all the types of PLA filters subjected to acetone pretreatment with different drying times. Strain was measured using an Instron ElectroPuls E3000-LT Series Test Instrument (Instron, Norwood, MA, USA), and the tensile strength of each sample at the point of breakage was measured. The 3D-printed PLA and the PLA subjected to acetone pre-treatment with drying times of 0, 60, and 1440 min. were used, and the displacement rate in the tensile tests was controlled to 10 mm/min. Three specimens representing the three types of filters prepared herein were tested.

### 2.5. MB Adsorption Experiment

Batch adsorption experiments were conducted to examine the potential of the PLA and GO/PLA filters. First, the chemically etched PLA with NaOH or acetone as well as the GO doped on pre-treated PLA filters were initially used for the removal of MB. A total of 0.2 g of each prepared filter was dropped in 25 mL of aqueous MB solution (50 mg/L), and the solution pH was adjusted to 6.5 by using 0.1 M HCl, 1 M HCl, or 1 M NaOH. The mixture was continuously agitated for 24 h in a vertical shaker operated at 40 rpm. The supernatant obtained after filtering the mixture through a 0.45-μm polyether–sulfone (PES) syringe filter was analyzed using an ultraviolet-visible (UV-Vis) spectrophotometer (Genesys 50, Thermo Scientific Co., Oxford, UK) at 662 nm to identify MB. To examine the adsorption kinetics, 0.8 g of the filter (approximately equal to 4 filters) was added to 25 mL of a 50-mg/L MB solution. The mixture was agitated for 0, 10, 30, 60, 120, 240, 360, and 1440 min. Adsorption isotherms were plotted in the MB solution concentration range of 10–70 mg/L by adding 0.2 g of the adsorbent.

The quantity of adsorbed MB and the MB removal rate were calculated using Equations (1) and (2) [30], respectively.

(1) $$q_{e}\;\left(\frac{\mathrm{mg}}{g}\right)=\left(C_{0}-C_{e}\right)\times \frac{V}{M}$$

(2) $$\mathrm{Removal}\;\mathrm{rate}\;(\%)=\frac{\left(C_{0}-C_{e}\right)}{C_{0}}\times \;100\;\%$$

where *C*_0_ (mg/L) is the initial concentration of the MB solution, and *C_e_* (mg/L) is the equilibrium MB concentration after the completion of adsorption. *V* (L) is the volume of the solution, and *M* (g) is the weight of the filter used in each batch experiment.

Adsorption kinetics were studied using Equations (3) and (4) [31,32].

(3) $$q_{t}=q_{e}\left(1-e^{-k_{1}t}\right)\;\;\;\;$$

(4) $$q_{t}=\frac{k_{2}q_{e}{}^{2}t}{1+k_{2}q_{e}t}$$

where *q_t_* is the amount of adsorbed adsorbate at any time *t* (mg/g), *q_e_* is the equilibrium concentration (mg/g), *k*_1_ is the first-order rate constant (1/min), and *k*_2_ is the second-order rate constant (g/mg·min).

Moreover, the intra-particle diffusion kinetic model (Equation (5) [33]) was used to investigate the adsorption mechanisms.

(5) $$q_{t}=K_{id}t^{\frac{1}{2}}+c$$

where *K_id_* (mg/g∙min^1/2^) is the intraparticle rate constant, and c (mg/g) is the thickness of the boundary layer formed in the first interval.

The obtained adsorption isotherm results were analyzed using the Langmuir (Equation (6)) [34] and Freundlich (Equation (7)) [35] isotherm models, which are expressed as follows.

(6) $$q_{e}=(q_{m}K_{L}C_{e})/\left(1+K_{L}C_{e}\right)$$

(7) $$q_{e}=K_{F}C_{e}{}^{\left(1/n\right)}$$

where *q_e_* is the amount of adsorbed adsorbate at equilibrium (mg/g), *q_m_* is the maximum adsorption capacity of the adsorbent (mg/g), *C_e_* is the equilibrium concentration of the adsorbate remaining in the solution (mg/L), and *K*_L_ is the Langmuir affinity constant (L/mg). *K*_F_ and *n* are Freundlich constants related to the adsorption capacity and adsorption intensity of the adsorbent, respectively.

## 3. Results and Discussion

### 3.1. Optimization of PLA Filter Pre-Treatment to Enhance GO Doping

#### 3.1.1. Effect of Solvent Types on GO Doping

**Figure 2:**
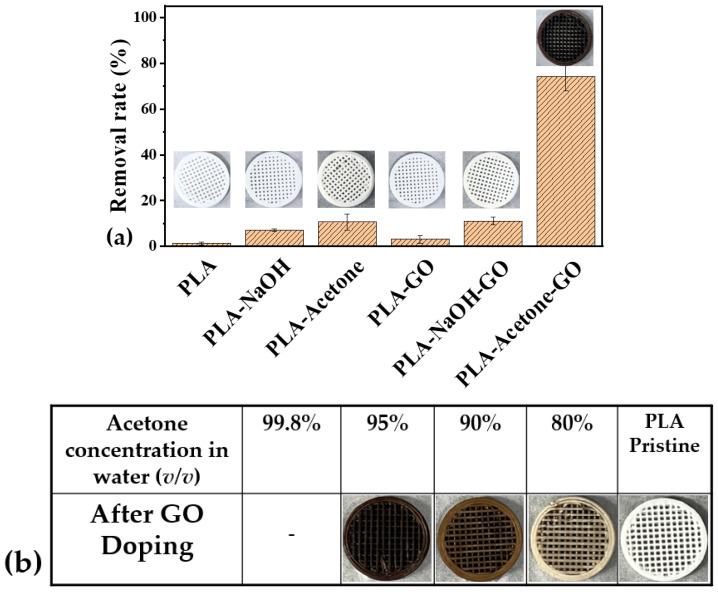
(**a**) Results of MB adsorption on PLA filters subjected to different surface treatments and (**b**) GO doped on PLA scaffolds treated with different concentrations of acetone (experimental condition: [MB]_0_ = 50 mg/L, pH = 6.5, reaction time = 24 h, average filter weight for MB adsorption = 0.2 mg).

The effects of different types of solvents on the PLA pre-treatment process were examined before GO doping by conducting a preliminary MB adsorption experiment, as depicted in Figure 2a. In this experiment, the 3D-printed PLA filter, not subjected to any pre-treatment, adsorbed only 1.26% of the MB in the solution. In the case of the PLA filter pre-treated with 5N NaOH solution, this number improved slightly to 7.07%. After the alkaline treatment, the PLA surface, which is relatively hydrophobic with water contact angles of 75–85° [36], was modified owing to the hydrolysis of the ester linkages in the PLA backbone chains and the formation of carboxylic and hydroxyl end groups in the polymer chains, resulting in improved MB adsorption on the PLA-NaOH filter [37]. Interestingly, a greater amount of MB from the solution was adsorbed on the PLA filter treated with acetone (PLA-acetone). Initially, the PLA scaffold was treated with undiluted acetone. However, this treatment softened the PLA scaffold and made it more flexible. Eventually, the filter became degraded and unable to retain its shape, which is consistent with the results reported elsewhere [21,38]. On the other hand, a diluted acetone solution with a concentration lower than 90% (wt.% in water) was not effective for GO doping compared to the case of 95 wt.% of acetone, as shown in Figure 2b. Therefore, we used 95% acetone in water (*v*/*v*) for acetone treatment of the PLA scaffold for 24 h, and the resulting PLA-acetone filter was able to adsorb 10.63% of the MB from the solution. In comparison to the PLA filter, the PLA-NaOH, PLA-acetone, GO-doped PLA filter subjected to either NaOH or acetone pre-treatment adsorbed higher amounts of MB, while MB adsorption on the GO-doped on un-treated PLA filter changed negligibly. Particularly, the GO-doped PLA filter subjected to acetone pre-treatment (GO-PLA) removed 74.21% of the MB in the solution. Additionally, the color changes in the PLA filters after different pre-treatment methods indicated that GO was uniformly distributed on the surface of the PLA-acetone filter. The chemical etching of the PLA scaffold with acetone is more strongly influenced by the surface modification of PLA for GO doping relative to the alkaline treatment. Thus, the 3D-printed PLA scaffolds were pre-treated with acetone to modify their surfaces, enhancing the adhesion between the printed PLA and nanoscale GO.

#### 3.1.2. Effect of Drying Time after PLA Pre-Treatment with Acetone

**Figure 3:**
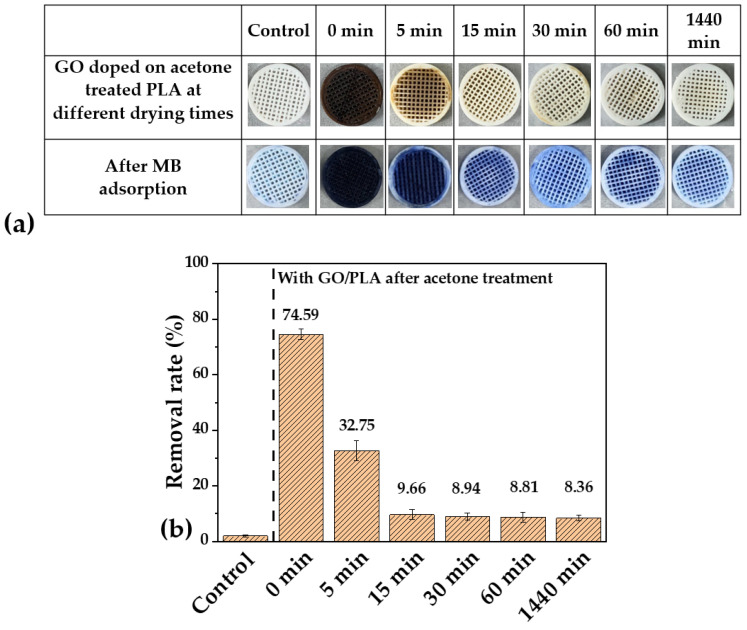
(**a**) Changes in the degree of GO doping with drying time after acetone treatment and (**b**) MB removal rate of GO/PLA filters with different drying time after acetone treatment. The experimental conditions were as follows: [MB]_0_ = 50 mg/L, reaction time = 24 h, weight of added PLA and GO/PLA filters = 0.2 g, and initial pH = 6.5.

The effect of drying time (at room temperature) after acetone pre-treatment was examined. Figure 3a shows the effects of drying time on GO doping on PLA pre-treated with acetone. Compared to the untreated PLA, chemical etching of the PLA scaffold with acetone and no drying time led to the most uniform distribution of GO on the PLA surface. As the drying time increased from 5 min to 1440 min, the effectiveness of GO doping decreased relative to that of the un-dried GO/PLA or GO doped immediately on PLA after acetone treatment, as indicated by the color changes in the figure. The GO/PLA filters were dried for different durations ranging from 0 min to 1440 min for MB adsorption, and the results are presented in Figure 3b. The undried GO/PLA filter was clearly the most effective for adsorbing MB from the solution (almost 74.6% removal), and the MB adsorption rates of the GO/PLA filters decreased as the drying time increased from 5 to 15 min; the adsorption rate did not change for drying times longer than 30 min.

**Figure 4:**
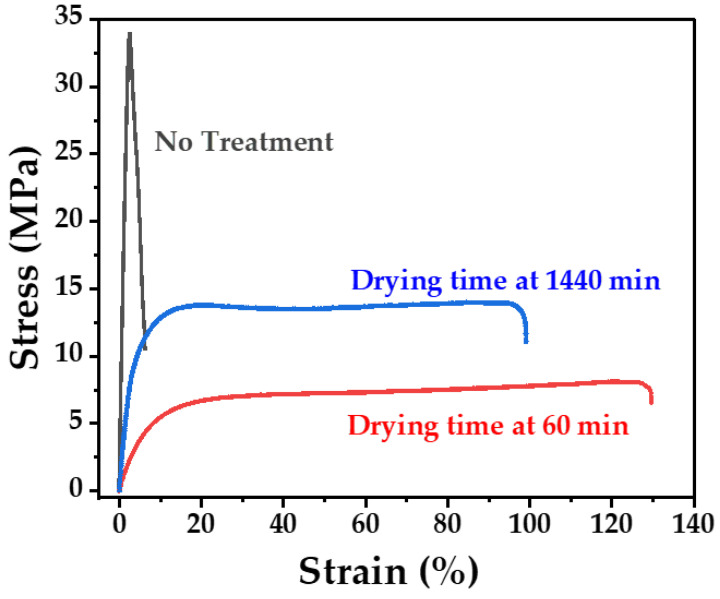
Stress-strain curves of PLA filaments for each drying time after pre-treatment with acetone.

To elucidate the reason for the decrease in the amount of GO loaded on the PLA scaffolds after acetone treatment with increasing drying time, the mechanical properties of PLA were examined by measuring its tensile strength. The test results are presented in Figure 4. The un-treated PLA scaffold exhibited brittleness owing to its inherent material properties relative to the acetone-treated PLA scaffolds, its tensile strength at yield and elongation at break were 33 MPa and 2.5%, respectively. However, the tensile strength of the GO/PLA filter dried for 1440 min and decreased to 13 MPa, and that of the GO/PLA filter dried for 60 min further decreased to 9 MPa. Meanwhile, the elongation at break values exhibited a reverse trend, improving from 94% to 127% with increasing drying time. These considerable changes in the tensile property of the PLA scaffolds after acetone treatment with different drying times can be explained by the fact that acetone dissolution partly changes the molecular structure of PLA and weakens the interaction between its molecular chains, resulting in decreased tensile strength and the induction of a rubbery texture [39]. Thus, acetone plays a role as a plasticizer, which leads to the swelling of the polymeric PLA filament, increasing its flow and flexibility [40] and eventually enhancing the GO doping on the PLA. Moreover, the effect of acetone as a plasticizer decreased as the acetone evaporated with respect to the drying time. The previous study reported by Coppola et al. (2019) [41] showed the use of acetone vapor for a post-processing surface smoothing treatment process. Hence, the surface modified PLA with acetone with drying time became smoother, and thus, the amount of loaded GO might become lower by increasing after the acetone treatment with increased drying time.

Finally, the enhanced motility of the PLA molecular chains can improve GO doping on the PLA surface, and without drying, the mechanical properties of PLA can be retained such that the resulting structure can function as an adsorbent filter. Based on the results, the 3D printed PLA scaffolds can be pre-treated under optimal conditions (95% acetone in water (*v*/*v*) for 24 h soakage prior to GO doping), and GO doping can be conducted without the drying time of acetone.

### 3.2. Confirmation of GO Doping on Chemically Pre-Treated PLA under Optimized Pre-Treatment Conditions

**Figure 5:**
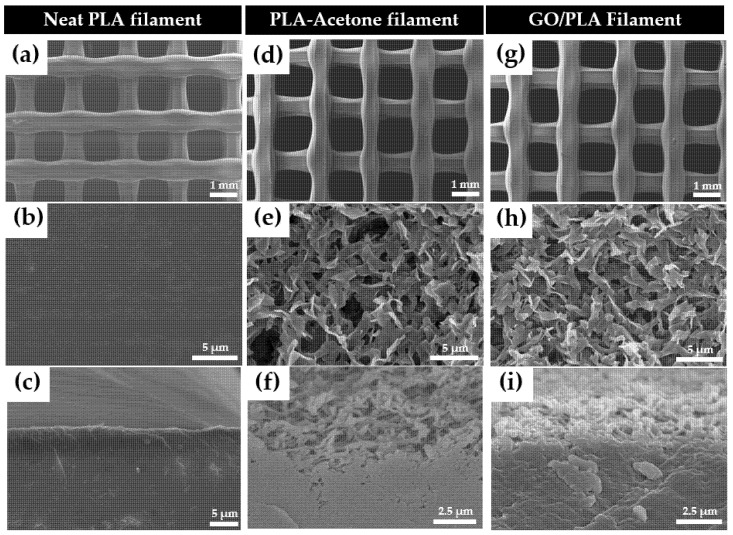
Surface morphologies of pure PLA filament (**a**–**c**), chemically etched PLA-acetone after acetone treatment (**d**–**f**), and GO/PLA (**g**–**i**). The bird eye view of filter (**a**,**d**,**g**), detailed view of surface (**b**,**e**,**h**), and detailed view of the cross-section of each filament (**c**,**f**,**i**) were provided.

Figure 5a–c show the surface morphology of the 3D-printed pristine PLA and how the extremely smooth surface structure of PLA changed after acetone treatment. The increased surface roughness of PLA can be observed in Figure 5d–f, and it indicates that the structural morphology of the PLA changed because of the acetone pre-treatment. After GO was doped on the chemically treated PLA, GO layers were formed, as can be observed in Figure 5g–i. Thus, GO loading on the rough surface of the acetone-treated PLA increased after 0 min of drying time.

**Figure 6:**
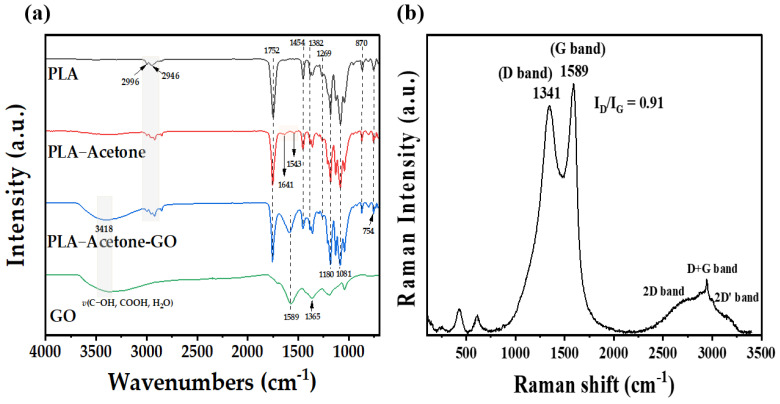
(**a**) FTIR spectra of PLA, PLA-acetone, PLA-acetone-GO (GO/PLA), GO, and (**b**) Raman spectrum of GO/PLA.

The GO/PLA filters prepared by following the optimized pre-treatment process (i.e., 95% acetone, 0 min drying time) were used to determine the characteristics of GO by means of FTIR and Raman spectroscopy. Figure 6a shows the infrared spectra of PLA, PLA-acetone, PLA-acetone-GO (GO/PLA), and GO. The peak assignments of neat PLA are shown in Figure 6a, and they are consistent with the values reported in the literature [42]. Infrared bands can be observed in the region between 2996 and 2946 cm^−1^, and they are ascribed to the asymmetric *v*(CH_2_) and symmetric *v*(CH_2_), which are readily recognizable in the spectra of PLA-acetone and GO/PLA [43]. The bands at 1454 and 1382 cm^−1^ correspond to bending vibrations of the methyl group (-CH_3_) in PLA [26]. The sharp, intense peaks at 1752, 1180, and 1081 cm^−1^ in the PLA spectrum are assigned to the C=O stretching vibrations of the carbonyl group and the C-O-C present in the ester group [26], and the presence of these characteristic peaks in the spectra of PLA-acetone indicates that the structure of PLA can be maintained even after acetone treatment. Interestingly, two new peaks at 1641 and 1543 cm^−1^ associated with C-O stretching and O-H bending vibrations, respectively, were present in the spectra of PLA-acetone, indicating that the carboxylic group on PLA was formed after acetone treatment. A small peak appeared at 754 cm^−1^, corresponding to the bending vibration of C=O, and the peak at 870 cm^−1^ was ascribed to the C-COO interaction within PLA. The FTIR spectrum of the GO shows O-H stretching vibrations at approximately 3400 cm^−1^, arising from the hydroxyl groups on the GO surface, while the bands at 1589 and 1365 cm^−1^ are ascribed to C=O and C-O stretching in the carboxylic groups, respectively. These peaks confirmed the chemical structure of GO.

The Raman spectrum of GO/PLA, shown in Figure 6b, confirms the formation of GO on the 3D-printed PLA surface. The D, G, and 2D bands characteristic of graphitic materials were observed, as reported in a previous study [44]. The obvious D band at 1341 cm^−1^ was associated with the out-of-plane vibrations in the graphitic lattice, and it was, therefore, related to the presence of disordered carbons in defects inside the graphene structure, while the G band at 1589 cm^−1^ was attributed to the in-plane stretching of the sp^2^-bonded carbon in the graphene [45,46]. The intensities of the D and G bands (I_D_^/^I_G_) indicate the density of defects, degree of disorder, and average size of the sp^2^ domains [5,9,47]. The higher I_D_^/^I_G_ ratio of 0.91 detected in the GO spectrum indicates that the GO underwent rigorous exfoliation and oxidation [48], and oxygen functional groups were present on it. Additionally, the 2D band at 2696 cm^−1^ indicated the presence of a graphene multi-layer, but the intensity of the 2D band was low.

**Figure 7:**
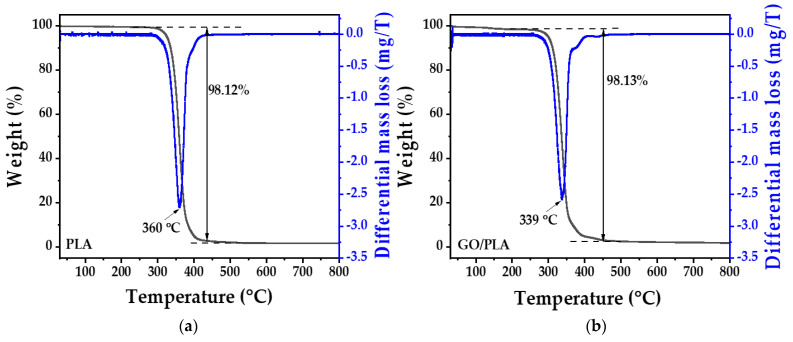
TG and DTG curves of (**a**) PLA and (**b**) GO/PLA.

The thermal stability of the printed 3D PLA filter and GO/PLA was examined using TGA. As shown in Figure 7, in the case of pristine PLA, major weight loss occurred at 360 °C, indicating its decomposition with a total mass loss of 98.12%. By contrast, the TGA curve of GO/PLA revealed one weight loss event at 339 °C, and the lowered decomposition temperature was attributed to the reaction between GO and PLA [43]. Unexpectedly, the GO peak was not observed, or it could have merged with the PLA peak, although GO decomposition was expected to occur at approximately ~258 °C owing to the removal of the oxygen-containing groups of GO [49]. The reason may be associated with the small amount of GO loaded on the PLA based on the insignificant difference in mass loss between PLA and GO/PLA.

To compute the actual mass of the GO loaded on PLA, the mass of the dry residue was obtained after the samples were heated to 800 °C. By using this value and the known percentage mass loss of PLA, the amount of GO doped on PLA was calculated as 0.3 mg on a dry basis. Considering the GO loading of 10 wt.%, the calculated GO doping on the chemically etched PLA filter was 2.16%.

**Figure 8:**
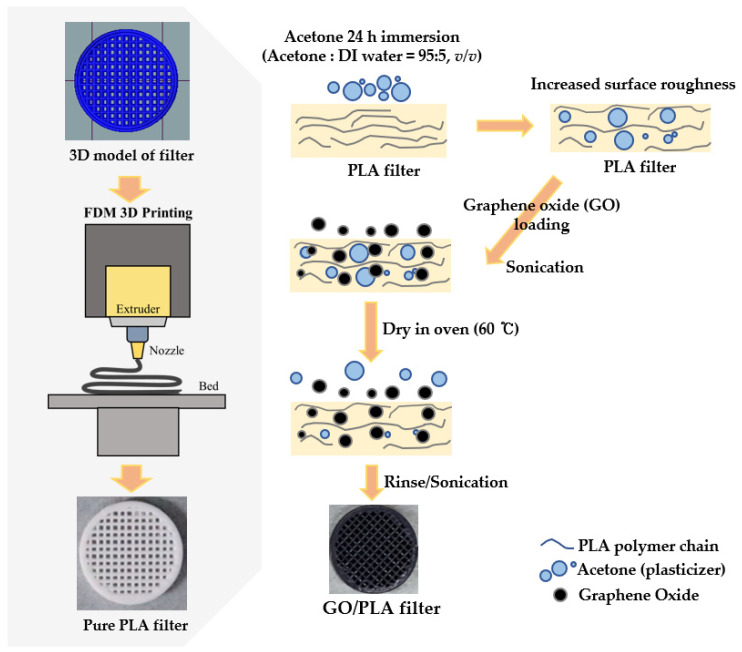
Schematic diagram of PLA fabrication with acetone pre-treatment to enhance GO doping.

Based on the obtained results, the schematic diagram presented in Figure 8 illustrates the optimized FDM 3D printing fabrication process for GO doping on the chemically etched PLA surface. After surface modification of the PLA with 95% acetone, the smooth surface of PLA becomes rough, and its tensile strength decreases, resulting in effective GO doping on the PLA surface. The GO-doped PLA filter (GO/PLA) was dried in an oven at 60 °C for 60 min, and any residual acetone or undoped GO particles on the PLA surface were removed by rinsing and sonication.

### 3.3. MB Adsorption Experiment

#### 3.3.1. Kinetics

**Figure 9:**
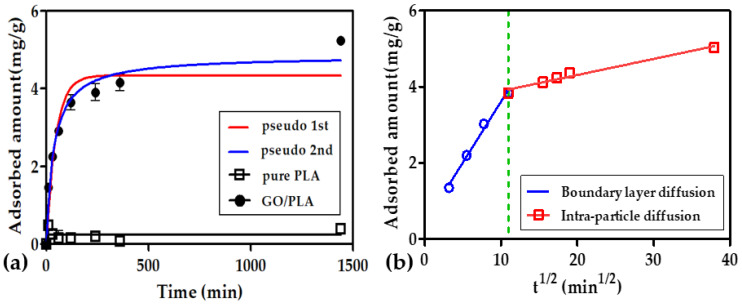
(**a**) Kinetic adsorption model and (**b**) intra-particle diffusion model for the adsorption of methylene blue on the GO/PLA filter (experimental conditions: [MB] _0_ = 50 mg/L, reaction time = 0–1440 min, weight of added GO/PLA for MB adsorption = 0.8 g, volume = 100 mL).

The characterized GO/PLA filter treated subjected to optimized pre-treatment has already proven its potential in terms of effective MB removal from a solution. Given the initial MB adsorption rate of the GO/PLA filter, a kinetic study of MB adsorption on the GO/PLA was performed. According to Figure 9, the amount of MB adsorbed on the PLA increased negligibly, whereas the GO-doped PLA filter adsorbed MB from the solution slowly and reached the equilibrium concentration within 360 min, indicating that a slow adsorption process is to be expected.

To explain the adsorption process in detail, two kinetic models, namely, the pseudo-first-order and pseudo-second-order models, were applied to the experimental data as shown in Figure 9a. The obtained data are summarized in Table 1. As illustrated by the high correlation coefficient (*R*^2^) values, the pseudo-second-order model yielded a better fit than the pseudo-first-order model, suggesting that the chemical reaction in this study significantly governed the rate-controlling step. The similar kinetic study of MB adsorption by graphene showed the best fit in pseudo-second-order mode [50].

The intra-particle diffusion model proposed by Weber and Morris (1962) [33,51] was applied in this study to determine the diffusion mechanism in the rate-determining step of the sorption process on a porous adsorbent. As depicted in Figure 9b, two main steps (i.e., an initial curve and a linear relationship) were observed. According to a previous study [52], the initial curve can be assigned to bulk diffusion through external surface adsorption or instantaneous adsorption, whereby the transport of dye molecules through the boundary occurs owing to the random motion of individual molecules. By contrast, the linear process is related to intra-particle diffusion. Since this line does not pass through the origin, the adsorption rate is not limited by pore diffusion during the initial periods, and the chemical reaction limits the adsorption rate in the early stages.

#### 3.3.2. Isotherms

**Figure 10:**
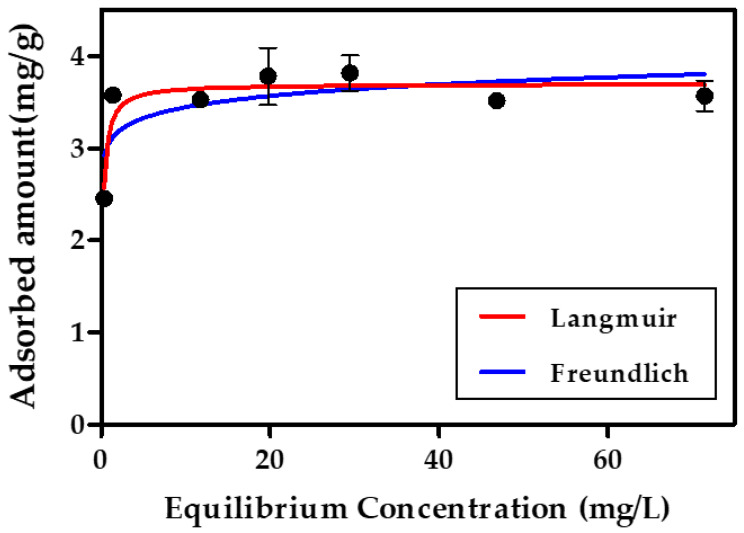
MB isotherm of GO/PLA filter obtained under the following experimental conditions: [MB]_0_ = 0.37–71.5 mg/L, weight of added GO/PLA adsorbent for MB isotherm = 0.2 g, volume = 25 mL. Black dots represent the raw data which were used for fitting either Langmuir or Freundlich model.

It is necessary to understand the adsorption isotherms to ascertain the adsorption mechanisms of the system considered in this study. Certain parameters obtained from the equilibrium equation indicate the surface properties of the GO/PLA adsorbent and its adsorption capacity for MB, leading to predictions of the maximum equilibrium adsorption. Among the several available isotherm models, the Langmuir and Freundlich adsorption models were applied to investigate the sorption mechanisms of MB molecules. By using these models, the mechanisms of MB uptake on GO/PLA were elucidated (presented in Figure 10), and the determined equilibrium parameters are summarized in Table 2. The equilibrium data for MB fit better to the Langmuir isotherm model with higher *R*^2^ values than to the Freundlich isotherm model. A number of works have observed similar phenomena on the adsorption of MB onto graphene [50], GO/MgO [53], and GO/Agar [54]. This means that the MB molecules were strongly attracted to the surface, and mostly mono-layer adsorption occurred. Moreover, once adsorption has occurred at specific sites within the adsorbent, no further sorption occurs at those sites. The surface housed specific homogeneous sites, and all the vacant sites had the same size and shape.

Furthermore, the dimensionless constant, that is, the separation factor (*R_L_*) of the GO/PLA filter, was calculated using Equation (8) [42]:

(8) $$R_{L}=\frac{1}{1+K_{L}C_{0}}$$

where *C*_0_ is the highest initial adsorbate concentration (mg/L), and *K_L_* is the Langmuir constant (L/mg). The *R_L_* value indicates whether the isotherm shape is un-favorable (*R_L_* > 1), linear (*R_L_* = 1), favorable (0 < *R_L_* < 1), or irreversible (*R_L_* = 0). The *R_L_* value in this study was 0.0023, indicating that MB adsorption on the prepared adsorbents was favorable.

The Freundlich adsorption isotherm as expressed in Equation (7) is an empirical equation that is used to describe heterogeneous systems [55]. It was applied to interpret the adsorption data of GO/PLA, whereby the magnitude of the exponent 1/*n* indicates the favorability of adsorption. The value of *n* in this study was 19.87, indicating extremely favorable adsorption (*n* > 1).

### 3.4. Environmental Implications of 3D-Printed GO/PLA

Recently, 3D-printed objects are being used in diverse environmental applications, especially in water treatment processes for pollutant removal. A few examples of 3D-filters printed using different types of printers (e.g., FDM, DIW, and DLP) are listed in Table 3. Previous studies have demonstrated that 3D printing technology is suitable for manufacturing low-cost filters through doping or in situ growth on the surface of support polymers [56,57,58,59]. Moreover, with the use of direct ink writing (DIW) printers, the desired composite materials can be printed directly through the nozzle by mixing the raw adsorbent materials together with binders [60]. In this study, the 3D-printed GO/PLA filter was customized through chemical etching of PLA to enhance GO doping on the PLA surface. Compared to other printing techniques, an FDM 3D printer with a simple, quick surface modification scheme was applied, and efficient MB removal was achieved through adsorption. In addition, the use of non-toxic chemicals for PLA surface modification is another benefit of the proposed 3D-printed filter. Thus, this study shows the potential of a 3D-printed GO/PLA filter as an efficient adsorbent for the removal of contaminants from water.

The adsorption capacity of the designed and fabricated 3D-printed GO/PLA filter for MB removal from solutions was compared to that of other adsorbents reported in previous studies (Table 4). The typical powdered carbonaceous adsorbents exhibit favorable adsorption abilities for MB removal from solutions [50,61]. Moreover, the effective adsorption capacities of GO-based adsorbents in the form of beads [62,63] and fibers [64] are notable. Interestingly, in the case of GO aerogels, cylindrical, porous, and structured GO-based adsorbents enhanced the MB adsorption capacity [65], suggesting that adsorbent geometry should be considered. In this study, a cylindrical 3D-printed GO/PLA filter was fabricated, and its MB adsorption capacity was 4.84 mg/g (equal to 200 mg/g based on the measured GO wt.%), which is similar to the adsorption performance of powdered and 2D adsorbents and slightly lower than that of GO aerogels. The lower adsorption capacity of the GO/PLA filter can be attributed to the small amount of GO doped on the PLA surface, and thus, rational design and fabrication methods should be developed in the future to enhance the adsorption performance of the 3D-printed lattice structure adsorbents.

## 4. Conclusions

(i) In this work, a 3D-printed GO-based filter, which could be used as an excellent adsorbent, was fabricated for the removal of cationic dyes from aqueous solutions.
(ii) PLA was selected as the matrix phase, and it was chemically treated with either a strong alkali (5N NaOH) or acetone. As compared with NaOH treatment, acetone treatment was an effective approach. After treatment with 95% acetone, the smooth surface of PLA became rough. FTIR analysis confirmed the presence of new peaks associated with carboxylic acids and hydroxyl groups, indicating changes in the structural properties of PLA after acetone treatment. In addition, its tensile strength decreased. Thus, the chemical etching of PLA with acetone enhanced GO doping on the PLA surface, leading to increased adsorption of MB from aqueous solutions.
(iii) The 3D-printed GO/PLA filter fabricated under the established optimal pre-treatment conditions was used to remove cationic MB molecules, and the pseudo-second-order model provided the best fit, indicating that the chemical sorption was the main reaction involved in the process. The isotherm model confirmed that MB adsorption occurred on the GO/PLA filter through mono-layer sorption.
(iv) This study highlights the importance of surface modification in promoting the hydrolysis of PLA for enhancing GO doping with the aim of increasing MB adsorption in aqueous solutions. Furthermore, the application of 3D printing technology helps with the development of adsorbents with different geometrics and lattices, which will open up a new window for the fabrication of 3D-structured adsorbents for use in water treatment processes.
(v) Based on the optimized pre-treatment strategies of PLA, different types of materials can be further applied to the pre-treated surface of PLA. Additionally, the optimized pre-treatment strategies can be further tested on the surface of PLA with different geometries in the future.

## Figures and Tables

**Table 1:** Parameters of the pseudo-first-order, pseudo-second-order, and intra-particle diffusion models for MB adsorption on a GO/PLA filter.

	Pseudo-First-Order Equation			Pseudo-Second-Order Equation		
	*k*_1_ (min^−1^)	*q_e_* (mg/g)	*R* ^2^	*k*_2_ (g/mg·min)	*q_e_* (mg/g)	*R* ^2^
Methylene Blue	0.02157	4.341	0.9098	0.005718	4.843	0.9611
	Boundary layer diffusion			Intra-particle diffusion		
	*K_i_*_1_		*R* ^2^	*K_i_*_2_		*R* ^2^
Methylene Blue	0.3200		0.9908	0.04218		0.9743

**Table 2:** Langmuir and Freundlich isotherm constants and values of MB adsorption on GO/PLA.

	Langmuir Isotherm			Freundlich Isotherm		
	*q_m_* (mg/g)	*K_L_* (L/mg)	*R* ^2^	*K_F_* (mg/g)	*n*	*R* ^2^
Methylene Blue	3.698	6.070	0.7647	3.068	19.87	0.4662

**Table 3:** List of 3D-printed adsorption filters for dye adsorption.

Name	3D Printing Type	Printing Material	Active Adsorbents	Fabrication Method	Target Contaminants	Ref
PLA@GO/Chitosan (CS)	FDM	PLA	GO and CS	Sonication - lyophilization	Crystal violet	[56]
3DP-HPC@MOFs	Direct ink writing (DIW)	SiO_2_ based thixotropic inks	3DP-HPC@MIL-100(Fe)	Dip coating with Polydopamine	Methylene blue, rhodamine B, crystal violet	[57]
3D-MOF@Clay	Digital light Processing (DLP)	Resin	Cr-MOF and nanoclay composites (Cr-MOF@Clay)	Sonication—dip coating	Methyl Orange	[58]
In-situ grown ZnO nanosheets	FDM	PLA	ZnO nanoflakes	NaOH treatment	Methyl Orange, rhodamine-B, methylene blue	[59]
Cu-BTC/SA-GE mixed inks	Direct ink writing (DIW)	Sodium alginate and gelatin (SA-GE matrix) + CaCl_2_	Cu-MOF	-	Methylene Blue, methyl violet, malachite green, rhodamine B, auramine O	[60]
GO/PLA	FDM	PLA	GO	Acetone treatment—dip coating	Methylene blue	This work

**Table 4:** Summary of adsorbents for MB removal from solutions.

Sample Type	Available Adsorbents	Preparation Method	*q_m_* (mg/g)	Reference
Powder	Graphene	Following a modified Hummers method	153.85	[50]
	Graphene oxide	Following a modified Hummers method	714	[61]
Bead (1D)	CMC/GOCOOH (Carboxymethyl cellulose microbeads incorporated carboxylated graphene oxide)	CMC/GOCOOH composite in the form of micro-beads were prepared.	180.32	[62]
Filter (2D)	GO/CA (GO/cellulose acetate)	Using a sol-gel method, GO/CA fibers were prepared.	181.81	[64]
3D aerogel beads	GO-MMT/SA (GO-montmorillonite/ Sodium alginate)	GO-MMT/SA hydrogel beads were prepared.	150.66	[63]
3D aerogel beads	LEGA (Lysine and EDA double cross-linked graphene aerogel)	3D cylinder shape of GO aerogel was prepared.	332.23	[65]
3D printed (3D)	GO/PLA	Cylinder shape of GO/PLA was produced using FDM 3D printer.	4.84 (200 based on GO wt.%)	This study

## Data Availability

The data presented in this study are available on request from the corresponding author.
